# Supplementary material for: Unrecognised COVID-19 deaths in central Europe: The importance of cause-of-death certification for the COVID-19 burden assessment
Source: PLoS One. 2024 Jul 16;19(7):e0307194. doi: 10.1371/journal.pone.0307194 (PMC11251637; doi:10.1371/journal.pone.0307194)
Supplement: S1 Table — (DOCX) [file pone.0307194.s001.docx]

Supplementary Table S1: COVID-19 and contributing conditions used in the analysis

| COVID-19 and contributing conditions | ICD-10 codes |
| --- | --- |
| COVID-19 | U07.1-U10.9 |
| ***Comorbidities*** | |
| Infectious: well defined infectious and bacterial diseases | A00-B99 except A40-A41, A48-A49, B34, B97-B99 |
| Neoplasms: malignant neoplasms | C00-C97 |
| Benign neoplasms, in situ neoplasm and all other neoplasms | D00-D49 |
| Immune: certain disorders involving the immune mechanism | D80-D89 |
| All diseases of blood and blood-forming organs | D50-D77 |
| Disorders of thyroid gland | E00-E07 |
| Diabetes: diabete mellitus | E10-E14 |
| Obesity | E66 |
| Disorders of lipoprotein metabolism and other lipidaemias | E78 |
| All other endocrine, nutritional and metabolic diseases | E except E00-E14, E40-E46, E66, E78, E86,E87 |
| Dementia: organic, including symptomatic, mental disorders | F00-F09 |
| Disorders due to psychoactive substance use | F10-F19 |
| All other mental and behavioural disorders | F20-F99 |
| Alzheimer and other degenerative diseases of the nervous system | G30-G32 |
| All other diseases of the nervous system | G00-G14, G20-G26, G35-G99 |
| Diseases of the eye and adnexa, the ear and mastoid process | H00-H95 |
| Hypertensive diseases | I10-I15 |
| IHD: ischaemic heart diseases | I20-I25 |
| Diseases of arteries, arterioles and capillaries | I70-I79 |
| All other diseases of the circulatory system | I00-I99 except I10-I26, I46, I50, I60-I79 |
| Chronic lower respiratory diseases | J40-J47 |
| All other diseases of the respiratory system | J00-J99 except J09-J18, J40-J47, J80-J81, J96 |
| Diseases of liver | K70-K77 |
| Diseases of the digestive system except diseases of the liver | K00-K93 except K70-K77 |
| Diseases of the skin and subcutaneous tissue | L00-L99 |
| Osteopathies and chondropathies | M80-M94 |
| All other diseases of muscoskeletal system and connective tissue | M00-M99 except M80-M94 |
| Diseases of the genitourinary system except renal failure | N00-N99 except N17-N19 |
| Trisomies | Q90-Q92 |
| Congenital malformations, deformations and chromosomal abnormalities except trisomies | Q00-Q99 except Q90-Q92 |
| ***Complications*** | |
| Sepsis, other bacterial infections and viral agents | A40, A41, A48, A49, B34, B97-B99 |
| Malnutrition | E40-E46 |
| Dehydration: volume depletion, fluid disorders | E86, E87 |
| AMI: Acute Myocardial Infarction | I21 |
| Pulmonary embolism | I26 |
| Cardiac arrest, heart failure | I46, I50 |
| Cerebrovascular diseases | I60-I69 |
| Influenza and pneumonia | J09-J18 |
| Adult respiratory distress syndrome, respiratory failure | J80, J81, J96 |
| Renal failure | N17-N19 |
| Respiratory arrest | R09 |
| Shock & Systemic Inflammatory Response Syndrome | R57, R65 |
| ***Other conditions*** | |
| Pregnancy, childbirth and the puerperium | O00-O99 |
| Senility, cachexia | R54, R64 |
| Ill-defined and unknown causes of mortality | R96-R99 |
| All other symptoms, signs and abnormal clinical and laboratory findings, not elsewhere classified | R00-R99 except R09, R54, R57, R64-R65, R96-R99 |
