# Supplementary material for: Unrecognised COVID-19 deaths in central Europe: The importance of cause-of-death certification for the COVID-19 burden assessment
Source: PLoS One. 2024 Jul 16;19(7):e0307194. doi: 10.1371/journal.pone.0307194 (PMC11251637; doi:10.1371/journal.pone.0307194)
Supplement: Attachment — Submitted filename: Response-to-Reviewers.docx [file pone.0307194.s003.docx]

**To the Editor and the Reviewers of manuscript ‘Unrecognised COVID-19 Deaths in Central Europe: the Importance of Cause-of-Death Certification for the COVID-19 Burden Assessment’ (submission no. PONE-D-24-12758)**

Dear Editor, Dear Reviewers,

We would like to express our sincere thanks for considering the paper for publication and giving us the opportunity to revise it. The Reviewers devoted considerable effort to reading our paper, and their comments were relevant and valuable in improving the manuscript.

In response to the Editor’s and the Reviewers’ comments and suggestions, we have made several changes to the manuscript, each of which is explicitly addressed in our responses. We have modified our manuscript in a change-tracking mode for easy reference during the review process. In the following sections of this letter, we provide point-by-point responses (in italics) to the concerns raised by the Editor and Reviewers.

We firmly believe that the manuscript has been significantly improved by the invaluable input of the Editor and Reviewers. We would like to thank you again for the time you have taken to review this manuscript.

Kind regards,

The manuscript authors

**Journal Requirements:**

1. Please ensure that your manuscript meets PLOS ONE's style requirements, including those for file naming. The PLOS ONE style templates can be found at 
<https://journals.plos.org/plosone/s/file?id=wjVg/PLOSOne_formatting_sample_main_body.pdf> and 
<https://journals.plos.org/plosone/s/file?id=ba62/PLOSOne_formatting_sample_title_authors_affiliations.pdf>

*Response: We ensured that the manuscript complies with PLOS ONE's style requirements.*

2. Please note that funding information should not appear in any section or other areas of your manuscript. We will only publish funding information present in the Funding Statement section of the online submission form. Please remove any funding-related text from the manuscript.

*Response: This mention has been removed accordingly.*

3. Thank you for stating the following financial disclosure:

   "This publication was supported by the University of Warsaw under the Priority Research Area V of the "Excellence Initiative – Research University" programme."

Please state what role the funders took in the study.  If the funders had no role, please state: "The funders had no role in study design, data collection and analysis, decision to publish, or preparation of the manuscript." 
If this statement is not correct you must amend it as needed. 
Please include this amended Role of Funder statement in your cover letter; we will change the online submission form on your behalf.

*Response: We have added the recommended statement that the funder had no role in the study in the separate cover letter.*

4. In this instance it seems there may be acceptable restrictions in place that prevent the public sharing of your minimal data. However, in line with our goal of ensuring long-term data availability to all interested researchers, PLOS’ Data Policy states that authors cannot be the sole named individuals responsible for ensuring data access (<http://journals.plos.org/plosone/s/data-availability#loc-acceptable-data-sharing-methods>).

Data requests to a non-author institutional point of contact, such as a data access or ethics committee, helps guarantee long term stability and availability of data. Providing interested researchers with a durable point of contact ensures data will be accessible even if an author changes email addresses, institutions, or becomes unavailable to answer requests.

Before we proceed with your manuscript, please also provide non-author contact information (phone/email/hyperlink) for a data access committee, ethics committee, or other institutional body to which data requests may be sent. If no institutional body is available to respond to requests for your minimal data, please consider if there any institutional representatives who did not collaborate in the study, and are not listed as authors on the manuscript, who would be able to hold the data and respond to external requests for data access? If so, please provide their contact information (i.e., email address). Please also provide details on how you will ensure persistent or long-term data storage and availability.

*Response: We have provided non-author contact details: telephone and email to the secretary of our institute, and email to the chair of the ethics committee of our institute. The data will be stored on the internal drive of the university in accordance with the data confidentiality policy.*

5. Please include captions for your Supporting Information files at the end of your manuscript, and update any in-text citations to match accordingly. Please see our Supporting Information guidelines for more information: <http://journals.plos.org/plosone/s/supporting-information>.

*Response: This has been corrected accordingly.*

6. Please review your reference list to ensure that it is complete and correct. If you have cited papers that have been retracted, please include the rationale for doing so in the manuscript text, or remove these references and replace them with relevant current references. Any changes to the reference list should be mentioned in the rebuttal letter that accompanies your revised manuscript. If you need to cite a retracted article, indicate the article’s retracted status in the References list and also include a citation and full reference for the retraction notice.

*Response: The references have been double-checked. One reference was deleted because it appeared twice in the list of references. Two additional references have been added:*

*Bugger H, Gollmer J, Pregartner G, Wünsch G, Berghold A, Zirlik A, et al. Complications and mortality of cardiovascular emergency admissions during COVID-19 associated restrictive measures. Schäfer A, editor. PLoS ONE. 2020 Sep 24;15(9):e0239801.*

*WHO. Estimates of Excess Mortality Associated With COVID-19 Pandemic (as of 5 April 2023). Geneva: WHO; 2023.*

7. If applicable, we recommend that you deposit your laboratory protocols in [protocols.io](http://protocols.io) to enhance the reproducibility of your results. Protocols.io assigns your protocol its own identifier (DOI) so that it can be cited independently in the future.

*Response: Following this recommendation, we have published the protocol describing our method of analysis on protocols.io. In this letter, we do not provide the doi for reasons of confidentiality (double blind review). However, if our paper is published, we will link the paper to the protocol.*

**Reviewer #1:**

Thank you for this well-written and highly relevant article. I only have minor comments:

1. In the abstract, first sentence methods: I would add '(Germany)' behind Bavaria, also the sentence is missing a verb, e.g., 'Data from ... was used.'

*Response: Thank you, both issues have been corrected accordingly.*

2. Introduction: I would not consider traffic accidents as the risk factor but for example heavy traffic or something alike

*Response: Yes, this was a mental shortcut. We corrected into ‘heavy traffic’.*

3. Analytical approach: I did not quite get '...and the frequency expected if causes u and c were independent' but it might just be me, however if you do have any space left than just a bit more info would be great

*Response: Again, this was a mental shortcut. The term ‘independent’ was used in a statistical sense, meaning that the co-occurrence of comorbidity c and underlying cause u is not significantly different from the co-occurrence of comorbidity c and other underlying causes. The authors who proposed the Cause-of-Death-Association Indicator originally argued that this measure is used to ‘identify unexpectedly frequent associations’ between causes of death (Désesquelles et al. 2010, p. 773) and that ‘many associations are indeed more frequent than would be predicted by a random occurrence of the diseases’ (Ibidem, p. 786).*

*We modified our manuscript in the following way :*

*The sentence:* ‘*The CDAI is the ratio of the observed frequency of a given complication or comorbidity c reported together with the UCoD u, and the frequency expected if causes u and c were independent’ (page 8, lines 170-173)*

*was replaced by the sentence: ‘The CDAI is the ratio of the observed frequency of a given complication or comorbidity c reported together with the UCoD u, and the mean frequency of cause c among all underlying causes of death’*

*The sentence: ‘For deaths with COVID-19 as a comorbidity and non-COVID-19 UCoDs, the Contributing Cause of Death Association Indicator (CCDAI) was established, defined as the ratio of the observed frequency of a given underlying cause u when comorbidity c is present, and the frequency expected if cause u were independent of comorbidity c’ (page 8, lines 182-185)*

*was replaced by the sentence: ‘For deaths with COVID-19 as a comorbidity and non-COVID-19 UCoDs, the Contributing Cause of Death Association Indicator (CCDAI) was established, defined as the ratio of the observed frequency of a given underlying cause u when comorbidity c is present, and the mean frequency of cause u among all comorbidities’.*

4. For '... average distributions of deaths by age, ̅ and ̅,, were calculated for the studied populations' I did not quite understand how population/death structure was taken into account (again may be me) [everything else was good to follow and nicely described]

*Response: These average distributions of deaths by age were used to remove the effect of the different age structure of deaths by underlying cause and gender, as recommended by Désesquelles et al. (2010). In our study, the* $\bar{d}_{x}$ *and* $\bar{d}_{c,x}$*, are the algebraic averages of death counts (by age x and cause c) in five populations and in both years under analysis (Poland 2020 was included in this standardisation step). This is equivalent to looking at the age distribution of the total number of deaths in all countries and both years, since in the CDAI formula, the average death term* $\bar{d}_{x}$ *appears both in the numerator and in the denominator. The inclusion of these averages in formulas no. (1) and (2) means that in effect we obtain for each country, year, gender and age a ‘standardised’ frequency of occurrence of a specific association of causes, which may be compared accross countries, years and genders.*

*To explain this step in our method, we modified our manuscript in the following way (page 9, lines 199-201):*

*‘To allow comparability across countries, years and genders, the average distributions of deaths by age,* $\bar{d}_{x}$ *and* $\bar{d}_{c,x}$*, were obtained by taking the arithmetic average of the number of deaths in all countries and years under analysis, taking into account both genders.*

5. Results: Tab1. - as Polands 2020 data are not considered (in the total) I would put them in brackets or grey, also you do state in the data section what 'contributing' means: every mentioning (part I or II death certifcate) of Cov19 except as UCoD, but I would put it again below the table (makes it easier for the reader to follow); also it could be an option to include a joint category of any mentioning of Cov19 on death certificate, which would sum up column 3 and 4, as you are refering to this in the first paragraph below fig. 1 (took me some calculating to get there) (similarily this could be done for Tab. 3). also I do undstand Polands low value of 3‰ but would probably stick with % (again to not confuse the reader)

*Response: Thank you for all these suggestions. We have amended Table 1 accordingly: we have added the detailed note on what ‘contributing’ means, added another column presenting the totals of deaths with any COVID-19 mention, and replaced 3‰ with 0.3%.*

6. I would not mind a literature reference for the 'typical coronavirus complications'

*Response: In the abstract and Results, the phrasing ‘typical coronavirus complications’ (‘COVID-19 reported as a comorbidity’ section) was used to refer to the most frequent medical conditions reported in the death certificates we analysed. In Discussion, when summarising this result, we provided (in the original manuscript) the references to other studies that identified the same COVID-19 complications (pages 15-16, lines 397-403):*

*‘The study identifies the most important complications aggravating the progression of coronavirus disease and leading to death, such as sepsis, pulmonary embolism, influenza, pneumonia, ARDS and respiratory failure, which is in line with previous prospective cohort studies [40–47] and Multiple Cause-of-Death studies for Brazil, Italy, and the United States [24,28–31,48].’*

*We hope that these references are sufficient.*

7. 'cerebral infarction, Acute Myocardial Infarction, other cerebrovascular diseases' - I would stick with lower case (in any case consistent would be good)

*Response: We acknowledge the lack of consistency but the capital letters are used according to the medical literature standards, where Acute Myocardial Infarction is often referred to as AMI, Ischaemic Heart Disease as IHD, Adult Respiratory Distress Syndrome as ARDS and Systemic Inflammatory Response Syndrome as SIRS. In contrast, general categories such as cerebral infarction are not referred to as CI.*

8. 'was estimated by the WHO at between 94,500 and 105,700' I suppose this refers to the different WHO scenarios (as it is not visible in the table), you should probably add that info

*Response: These figures refer to low and high estimates, whereas Table 3 only includes the mean estimate. In the revised manuscript, we have clarified this in the following way:*

*– The heading of Table 3 includes a note that the numbers refer to the mean estimate, and*

*– The text describing Table 3 has been rephrased to (page 14, lines 345-346, new text in bold):*

*‘For Poland, surplus mortality in 2021 was estimated by the WHO* ***at 100,100 (mean estimate), ranging from 94,500 (low estimate) to 105,700 (high estimate), which is*** *slightly above the number of all COVID-19 mentions registered in that year.’*

9. Discussion: in the third paragraph you are refering to Polands excess deaths in 2020, whereas you are not looking at 2020 data beforehand, maybe stick with 2021 here as well? also I was not quite sure where those 67,000 excess deaths come from as they are not displayed in Tab. 3 (though it might be Ref. 50)

*Response: Yes, sorry for not making this clear in the original manuscript. The quoted 67 thous. excess deaths in 2020 come from the reference no. 50 (a report by the Ministry of Health of Poland) and to stress this, we modified the manuscript in the following way (pages 16-17, lines 423-427, new text in bold):*

*‘In these countries, the elevated mortality in the pandemic period should be attributed to other causes of death or to unrecognised COVID-19 cases, but the different diagnostic testing policies implemented by Lithuania and Poland make it difficult to draw broad comparisons^49^.* ***Nevertheless, a report from the Ministry of Health of Poland^50^ suggests that the number of unrecognised COVID-19 cases in that country was considerable in 2020, the year for which data on multiple causes of death are not available. According to this analysis,*** *67,000 excess deaths were reported in 2020, of which 43% were due to COVID-19 as the UCoD and 27% due to other UCoDs in coronavirus patients.’*

*Note that the Ministry of Health’s estimate of 67,000 excess deaths is similar to the WHO mean estimates shown in Table 3 (63,000), which we believe lends credibility to the Ministry's estimation method.*

10. I personally, would have expected at least a little elaborating on other sources of excess mortality (delayed health care access etc.) and even reductions in mortality (e.g. less traffic accidents) in the discussion, you briefly mention those in the beginning but never get back to this and it should at least be acknowledged that other things have happend during pandemic times and not all excess can or should (?) be diretly attributed to COVID-19

*Response: Such an acknowledgement was easily incorporated at the end of the discussion; we modified the manuscript as follows (pages 18-19, lines 464-479, new text in bold):*

*‘****During the COVID-19 pandemics, mortality increased far beyond the reported deaths from coronavirus disease, and several factors were responsible for this. Many studies suggest that health care systems were either overburdened or entirely reserved for people diagnosed with coronavirus [9–14], which contributed to postponement of treatment of chronic diseases or neglect of acute conditions, especially cardiovascular symptoms [52]. Another strand of research points to the long-term health consequences of COVID-related closure restrictions and the economic downturn: stress and mental health problems, the misuse of harmful substances, and related self-harm and accidents [16–18]. While the importance of these factors can be approximated by studying mortality from causes other than COVID-19, the scale of unrecognised coronavirus cases has rarely been explored due to lack of data and methods. The most recent study [23] showing that peaks in COVID-19 deaths were preceded at short time intervals by peaks of all-cause mortality in some regions of the United States is a novelty in this respect. This study shows that*** *despite the complexity of the pathophysiological process leading to death in the presence of SARS-CoV-2, the use of MCoD data and metrics allows us to overcome some of the ambiguity of certification practices and to assess the overall burden of COVID-19.’*

Reviewer #2: Good Manuscript, highlights the role of death certification in evaluating excess deaths, in this circumstance, occurring during COVID, and highlights areas where proper medical certification of death, would have identified these mortalities. In addition, this paper highlights the burden of COVID mortality, among persons with co-morbidities, particularly obesity, and cancer diagnosis. It is particularly useful in the assessment of death occurring in these populations, and particularly, development of hypotheses, on how to protect these vulnerable persons. It also addresses aspects of COVID, that may address potential areas of misinformation, eg vaccine hesitancy.

*Response: Thank you for your recognition of this study. We also believe that this work provides evidence of the importance of country-specific medical certification practices for COVID-19 identification and reporting to the national authorities and international organisations. Our conclusions can be extended to other areas of public health, particularly emerging diseases, where accuracy of cause-of-death reporting and speed of response are of high priority.*
